# Pedestrian Counting with Occlusion Handling Using Stereo Thermal Cameras

**DOI:** 10.3390/s16010062

**Published:** 2016-01-05

**Authors:** Miklas S. Kristoffersen, Jacob V. Dueholm, Rikke Gade, Thomas B. Moeslund

**Affiliations:** Visual Analysis of People Lab, Aalborg University, Rendsburggade 14, 9000 Aalborg, Denmark; mskr11@student.aau.dk (M.S.K.); jdueho11@student.aau.dk (J.V.D.); tbm@create.aau.dk (T.B.M.)

**Keywords:** computer vision, pedestrian counting, occlusion, thermal, infrared, stereo, 3D reconstruction, point cloud, tracking

## Abstract

The number of pedestrians walking the streets or gathered in public spaces is a valuable piece of information for shop owners, city governments, event organizers and many others. However, automatic counting that takes place day and night is challenging due to changing lighting conditions and the complexity of scenes with many people occluding one another. To address these challenges, this paper introduces the use of a stereo thermal camera setup for pedestrian counting. We investigate the reconstruction of 3D points in a pedestrian street with two thermal cameras and propose an algorithm for pedestrian counting based on clustering and tracking of the 3D point clouds. The method is tested on two five-minute video sequences captured at a public event with a moderate density of pedestrians and heavy occlusions. The counting performance is compared to the manually annotated ground truth and shows success rates of 95.4% and 99.1% for the two sequences.

## 1. Introduction

Pedestrian counting can provide important information for the purposes of setting prices in shops based on the number of potential customers passing by, adjusting personnel and opening hours, measuring the impact of shop events, designing marketing strategies aimed at increasing the number of customers in a limited time period, or ascertaining that all individuals are safely evacuated in emergency situations. The key data for all these activities are the number of people in the scene. Such a system has to work in indoor as well as outdoor settings 24 h a day. In outdoor applications, it is particularly difficult to use regular RGB (Red-Green-Blue) cameras due to changing lighting conditions and impossible to use them during dark nights. The thermal camera is an alternative sensor that has recently become popular for e.g., pedestrian detection [1]. The light-independent properties of this sensor make it very suitable for outdoor surveillance. By capturing the long-wavelength infrared radiation (LWIR), the image represents the thermal radiation range of the heat emission pattern of humans [2]. Humans’ body temperatures are often different from those of their surroundings, which makes it relatively simple to segment objects of interest.

Pedestrian counting is by now a well researched field that still provides several challenges. The main concern in pedestrian counting is occlusions, which occur especially in high density scenes. Other concerns include varying densities, differences in the appearance of pedestrians, and the unpredictable behavior of pedestrians [3,4]. The occlusion problem is often partially mitigated by placing the camera in the zenithal position (top-down view), although occlusions still occur at the peripheral areas of the image, especially when using a camera with a wide field of view [5]. The zenithal view is often used when counting at entry or exit points but is rarely an option in practice when monitoring public pedestrian streets.

In this paper, we take a different approach and suggest the use of a stereo setup of thermal cameras. This adds depth information about the scene, while preserving the advantages of thermal imaging.

### 1.1. Related Work

A stereo thermal setup is already seen in a number of applications e.g., sparse density matching [6], odometry [7], room mapping with low visibility due to smoke [8], and improving face recognition by fusing stereo, RGB, and stereo thermal [9]. Two fields that pay particular attention to it are pedestrian detection [10,11,12] and vehicle navigation [12,13,14,15].

As found in [16], a common ground is needed to compare different methods in the thermal modality. Recently, thermal databases have become available [17,18], but none of them use a stereo thermal setup, and a corresponding RGB video is rarely available for comparison. Thermal stereo introduces new challenges in calibration, when regular calibration boards might not suffice [19,20].

Various people counting solutions in the visible spectrum exist, generally categorized into direct and indirect approaches, and a number of comprehensive survey papers about them have been published [21,22,23,24]. The direct approach is detection-based, tracking each individual in the scene [25,26,27,28]. This method requires a strong segmentation, which struggles in the presence of occlusions. Survey papers of pedestrian detection methods can be found in [3,29,30]. This approach relies on tracking methods to associate detections between frames. Single object tracking is analyzed in [31], while [32] deals with multiple object tracking. The indirect approach, in contrast, is map-based, and estimates the number of pedestrians in the scene [33,34,35]. The estimates are less precise at varying densities and varying distances but are found to be more robust overall than the detection-based method.

Methods combining both the direct and the indirect approach show promising results. They detect groups rather than individuals and estimate the number of people contained in each group. This approach is seen in [36], where SURF (Speeded Up Robust Features) points are extracted and grouped into clustersusing a graph-based clustering algorithm. The number of people in a cluster is estimated using an *ϵ*-Support Vector Regressor with several parameters such as the distance to the camera and the density of features in the cluster. The regressor is trained using only 30–40 frames. The pedestrian count is calculated as an average value of the last *k* frames. This approach was further developed by [37], applying a three-frame difference algorithm to extract a binary mask containing movement. Only this region is searched for SURF features, which speeds up the process considerably. Features are clustered using an improved version of DBSCAN (Density-Based Spatial Clustering of Applications with Noise). Furthermore, a tracker is introduced, tracking clusters using a local Lucas-Kanade optical flow algorithm with a Hessian matrix.

A similar approach is found in [25], where FAST (Features from Accelerated Segment Test) and Tomasi-Kanade are used in a Bayesian clustering algorithm to detect individuals. Frame differencing is also used by [38] to extract interest regions based on motion. These regions are both used for learning an appearance model, and for tracking SIFT (Scale Invariant Feature Transform) features, thereby handling partial and full occlusions. In [39] they find FAST and SIFT to be inadequate, since only few features are found in the application of abnormal behaviour. Instead, a tracker similar to Lucas-Kanade is used to generate trajectories. The Lucas–Kanade tracker is also used to recognize activities between different views in [40].

RGB-based systems are sensitive to illumination changes and shadow inference. One way to overcome these challenges is to use two cameras in stereo [41]. Changes in illumination occur in both cameras; thus, they can still match features. Features are projected from the camera 3D coordinate frame down on a 2D street plane, where the height is used to filter shadow inference. Occlusions are thereby also limited in a top view. A kernel-based algorithm is applied to group feature points on pedestrians and a splitting algorithm is implemented to divide erroneously large clusters into two minor clusters.

### 1.2. Our Contribution

The ability to operate both day and night make a thermal stereo application an appealing solution to the challenge of counting pedestrians. In this paper, we investigate the reconstruction of 3D points in a pedestrian street with two thermal cameras. In addition, we propose an algorithm for pedestrian counting based on clustering and tracking of the 3D point clouds. The proposed system is evaluated on challenging real data with a high degree of occlusions captured at a public event.

## 2. Thermal Stereo Imaging

Three-dimensional reconstruction using stereo cameras is a well-studied topic that is typically approached with RGB cameras. Many of the recent stereo applications acquire depth data in a scene with factory-calibrated stereo rigs such as the Bumblebee [42]. The basic point of stereo imaging is to reconstruct a point located in world space by projecting the point to two image planes and finding the intersection of the two projection rays. Thus, two of the most important concepts of stereo imaging are correspondence between two images and triangulation, of which the former is the more challenging. The correspondence problem is solved for all pixels in most applications, which means that every pixel in an image is assigned a corresponding pixel in the other image of the stereo pair. The result is a dense disparity map or 3D reconstruction.

The principle of 3D reconstruction using two thermal cameras is theoretically identical to using RGB cameras. While solving the pixel correspondences can be used to extract dense depth information from the scene, it requires that the images are highly textured in all regions. This is not the case with thermal images, however, which means that there is a higher percentage of mismatches and thus a greater need for filtering. Another approach is to solve the correspondence problem for good features only. The sparse selection of data decreases the execution time when processing points, and it ensures a more accurate 3D reconstruction. Thus, a robust and efficient method for extracting, describing, and matching features is needed. This topic that has been researched for many years in the visible spectrum, but it has yet to be analyzed in-depth in the LWIR spectrum. Ricaurte *et al.* show that most well-known algorithms have same relative ranking in the thermal spectrum compared to the visible spectrum [43]. Mouats *et al.* choose Fast-Hessian detectors and FREAK (Fast Retina Keypoint) descriptors due to the low computational cost and relatively good performance [7]. Furthermore, they refer to a performance analysis of the methods in the LWIR spectrum, which, at the time of this study, was still under review. Hajebi and Zelek, on the other hand, choose phase congruency for detecting and describing features [6]. This method has a high computational cost, but it shows good results.

Generally, the scene in a pair of synchronized stereo images does not change much between the two images. This suggests the use of simple block matching around good features as an efficient and accurate way of solving the correspondence problem. However, this method assumes no change in viewing angle as it is not invariant to anything other than translation. This means that it might return false matches in different setup scenarios due to, among other things, baseline length, non-parallel cameras, and distance to pedestrians.

As an in-depth comparison of feature detection methods for LWIR images is still not available, we compare the performance of a number of well-known algorithms from the RGB domain on our training data. Table 1 compares the execution time, number of feature points, and percentage of matched features on ten different combinations of detectors and descriptors. All methods are tested using the default values of the OpenCV implementation. The performance of the methods are described relative to each other during 300 frames of the training data. The execution time is described as *short* if fewer than 30 milliseconds are used per frame and *medium* if it is between 30 and 200 milliseconds, leaving a group with a *long* execution time if more than 200 milliseconds are spent per frame. All methods that extract at least 100 features per frame belong to the *many* category of the *Features* column. *Medium* is between 30 and 100 features per frame, and *few* denotes fewer than 30 features. In the *Matched Feat.* column, a method is described as *high* if more than 30% of the extracted features are matched correctly. RANSAC is used to estimate the fundamental matrix, which is used to decide if a match is an in- or outlier. *Medium* is a score between 20% and 30%, and *low* is below 20%.

**Table 1:** Comparison of feature detectors and descriptors in the long-wavelength infrared radiation (LWIR) spectrum.

Detector	Descriptors	Execution Time	Features	Matched Feat. (%)
BRISK [44]	BRISK [44]	very long	very few	–
BRISK [44]	FREAK [45]	very long	very few	–
FAST [46]	BRIEF [47]	very short	medium	low
Shi–Tomasi [48]	FREAK [45]	medium	very many	very low
Shi–Tomasi [48]	SIFT [49]	medium	very many	high
Shi–Tomasi [48]	SURF [50]	medium	very many	very low
Oriented FAST (ORB) [51]	Rotated BRIEF (ORB) [51]	short	many	medium
DoG (SIFT) [49]	SIFT [49]	long	few	medium
Fast–Hessian (SURF) [50]	SURF [50]	long	medium	high
Fast–Hessian (SURF) [50]	FREAK [45]	long	medium	very low

As a compromise between execution time, number of features, and quality of features Shi–Tomasi detectors and SIFT descriptors are chosen to apply in this paper.

### Stereo Matching

For thermal images, the background often contains very little texture, so, as discussed earlier, matching points will be found only for foreground objects. To simplify the matching and reduce spurious detections, the first step in the algorithm is to remove the background from both views in every frame.

The background is estimated as the median for each pixel of a series of images, ensuring that the background is visible in more than half of the frames for each pixel. This assumption works in a pedestrian street with a continuous flow that is not too crowded. Alternatively, the background can be chosen as a frame with no pedestrians present. The estimated background is updated for each frame with a low learning rate from the input image. From this, it follows that, if any pedestrians stop in the scene, they will eventually be part of the background and thus excluded from the search for features. The choice of learning rate is thus a trade-off between vulnerability to changes in the temperature of the environment (e.g., a quick shower might temporarily decrease the temperature of the street) and pedestrians becoming part of the background.

**Figure 1:**
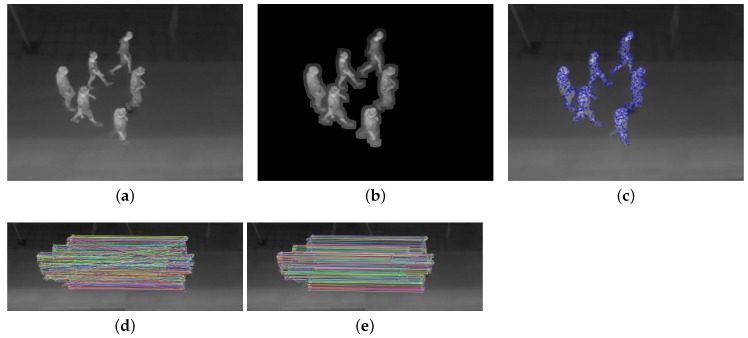
Example showing the extraction and matching of features in a thermal stereo image pair. (**a**) undistorted right input image; (**b**) applied foreground mask; (**c**) detected features; (**d**) matches; (**e**) matches with *y*-constraint. Note that (a)–(c) are performed for both the left and right images.

With the foreground mask defining regions in which to search for features it is possible to avoid potential mismatches. However, there is still a risk of mismatches as pedestrians appear very similar in thermal images due to low resolution and contrast. Thus, the leg of one pedestrian might look identical to the leg of another pedestrian. In order to remove these mismatches, we use the fact that two corresponding points must satisfy the epipolar constraint. In the specific case with approximately parallel cameras, as shown in Figure 1, the corresponding points can be assumed to have only a small deviation in the *y*-direction.

**Figure 2:**
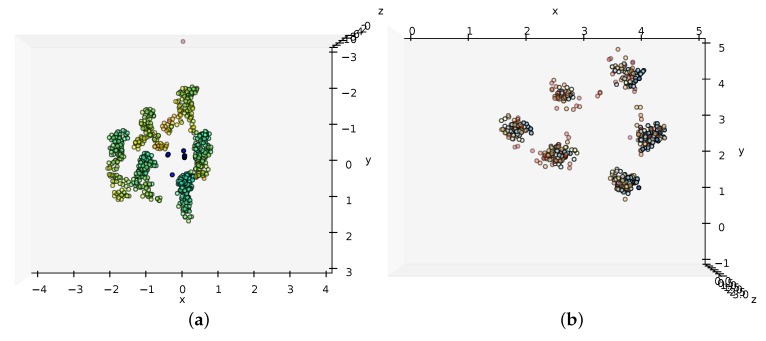
Example showing the reconstructed 3D points of the example shown in Figure 1. The points are colored according to their *z*-coordinate. (**a**) 3D points in the left camera frame; (**b**) top-down view of the 3D points in the street frame.

The corresponding points are used to reconstruct the 3D points with a linear triangulation method [52]. An example of the resulting 3D points is shown in Figure 2. In order to make the detection of pedestrians simpler, the points are rigidly transformed to the street frame using the method from [41] but with human features instead of markers.

## 3. Group Definition and Detection

**Figure 3:**
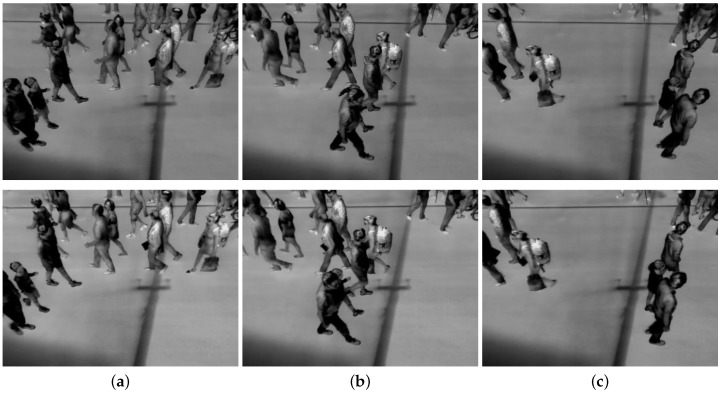
Example of pedestrians occluding each other. The upper row displays left camera images, while the bottom row displays right camera images. (**a**) is frame 0, (**b**) is frame 45, and (**c**) is frame 90. Note the two people entering the lower left side of the images in (a); in (b) the child is almost fully occluded, while she appears again in (c).

In a pedestrian street environment, it is often not possible to mount cameras in a zenithal position above the street. Other angles of view imply occlusions between pedestrians, making it difficult to separate individuals from one view. Including depth information can add information that is needed to solve ambiguous situations. However, a pedestrian still has to be visible in both views in order to be reconstructed in 3D. From this, it follows that if a pedestrian is fully or mostly occluded in one or both views, the person will not exist in the reconstructed scene. An example of this situation is shown in Figure 3.

Thus, pedestrians might disappear and appear in the scene as a consequence of occlusions, which places high demands on the tracking and counting module. One way to solve this problem is to track groups instead of individuals as seen in [53]. This allows for pedestrians to disappear temporarily but still belong to a group that is tracked correctly. Including an estimation of the number of members in each group makes it more robust against occlusions than when tracking each individual.

It is difficult to explain when a collection of pedestrians should be considered a group. One way to look at it is to use the spacing between pedestrians. This is precisely what the proxemics theory introduced by Hall [54] addresses. According to Hall, the social relations between pedestrians impact the physical distance between them. He also introduces some rough guidelines for spacing, which, in our case, can be used to define groups of people walking together, *i.e.*, pedestrians that are within 2.5 feet (≈ 0.8 m) (close phase of personal distance [55]) of each other are assumed to be together in a group.

The 3D points are clustered according to the distances defined by Hall in order to detect groups and the number of pedestrians in each of them. As we do not know the number of groups or pedestrians in the scene, we apply the hierarchical clustering algorithm DBSCAN [56]. This is a density-based algorithm that has two parameters controlling the termination of the algorithm. These two are distance Eps and number MinPts. In short, a point *p* belongs to a cluster if it has at least MinPts points within a distance of Eps, also referred to as $N_{Eps}\left(p\right)$. However, in order to handle border points and noise correctly the standard DBSCAN algorithm is as follows:

1. An arbitrary point *p* from the point cloud that is not part of a cluster or marked as noise is chosen.
2. If $N_{Eps}\left(p\right)>MinPts$ a cluster is started; otherwise, *p* is marked as noise and we jump to 4.
3. The points in $N_{Eps}\left(p\right)$ are pushed onto a stack *S*.
  (a) A point *q* is popped from the stack *S*.
  (b) If the point *q* is not part of a cluster, it is marked as part of the current cluster.
  (c) If (b) was true and $N_{Eps}\left(q\right)>MinPts$ the points of $N_{Eps}\left(q\right)$ are pushed onto the stack *S*.
  (d) (a)–(c) are repeated until the stack *S* is empty.
4. Steps 1–3 are repeated until all points are classified as either noise or part of a cluster.

Note that it is possible to visit points multiple times, which makes the algorithm less efficient. The algorithm also has a large memory footprint. For these and other reasons, several suggestions for improving the original algorithm have been proposed (for examples see [37,57,58]). However, as the point cloud in this application is small, the original version of DBSCAN is found to be sufficient.

### Procedure

Due to erroneous matching, some of the 3D points in the street frame will be spurious. Furthermore, some points will be placed outside the region of interest. The region of interest is defined as a 3D box such that points below a certain height are removed in order to avoid the inclusion of dogs, small prams, and other objects that are not as tall as pedestrians. The same goes for points that are above a certain height or outside a rectangle defined in the street plane. This leaves points from the waist up of adults in the image and points from the top of children’s heads, depending on how tall they are.

The remaining points are looped to check the number of points in their neighborhood in a DBSCAN manner. Those with an insufficient number of neighbors are removed, as they are assumed to be faulty matches. This procedure violates the DBSCAN definition of border points [56] and might remove some points that are not noise, but the harm that causes is so minor that it outweighs, in our opinion, the benefit of removing the noisy points.

**Figure 4:**
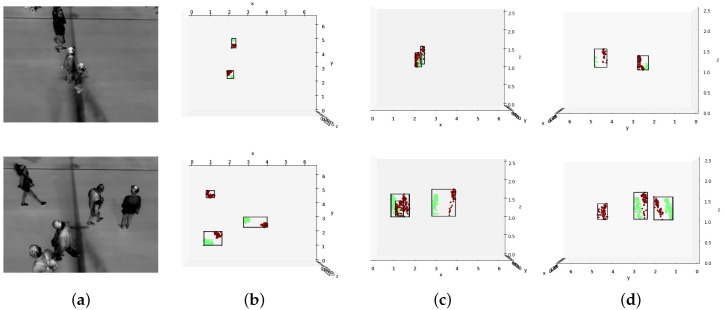
Examples of detected groups and pedestrians. The upper row presents one example, the bottom row another. Each group is shown within a bounding box and each pedestrian in the group is represented by a different color. (**a**) are the undistorted left input images; (**b**) are top-down views of the reconstructed scene; (**c**) are views from the base of the camera position; (**d**) are views along the street.

The 3D points are clustered using DBSCAN with an $Eps_{\mathrm{group}}$ and $MinPts_{\mathrm{group}}$ optimized for detecting groups of people. The resulting groups are further clustered with an $Eps_{\mathrm{pedestrian}}$ and $MinPts_{\mathrm{pedestrian}}$ optimized for detecting individual pedestrians. An example is shown in Figure 4. With a set number of pedestrians in each group, the groups can be described using simple measures like 2D street position (center of mass) and number of group members.

## 4. Tracking and Counting

It is necessary to track the detected groups in order to keep track of the number of pedestrians passing through the system. For this purpose, we apply the Kalman filter [59] as it efficiently handles multi-object tracking and noise filtering. The state vector of each filter consists of (x,y) coordinates, $\left(\dot{x},\dot{y}\right)$ velocities, and *n* number of group members. There are a total of five elements in the state vector. Of these five, only three are measured, namely (x,y,n).

For each frame, a matrix is solved consisting of the detected groups in rows and the predicted states of the filters in columns [60]. There are six scenarios:

1. A detected point does not belong to any track and thus creates a new tracker.
2. A detected point matches a track and is used to correct the state.
3. Two or more detected points match a track, which suggests a split of the track.
4. A detected point matches two or more tracks, which suggest a merge of the tracks.
5. A track has no matching points and is deleted after a number of consecutive frames without any assignments.
6. There are complex situations in which trackers are both suggested to split and merge.

A point and a track match if the detected point is within a certain distance of the predicted state of the filter. The distance is a global measure that is the same for all filters and uses the position of the centre of each group.

**Figure 5:**
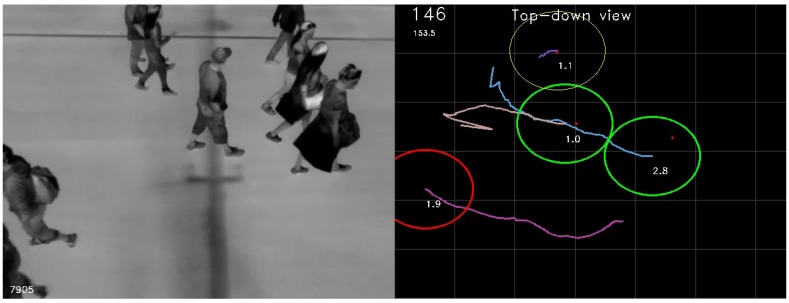
Example of the running system. The image on the left is the undistorted left input image; the image on the right is a top-down view of the street used to show the tracked groups. The number in the lower left corner of the left-hand image (7905) is the current frame number. The number in the top left corner of the right-hand image (146) is the current count of pedestrians that have walked by. The number just below that (153.5) is the current count without rounding. The center of mass of each detected group is shown with a small red dot. Each predicted state has a predicted number of group members shown as a small number (1.9, 1.1, 1.0, 2.8). The maximum allowed distance from the predicted positions is shown by circles in different colors and line thicknesses. Thin yellow means that the track is young and has a point in the current frame. Thin red (not shown in this case) is young and does not have a point. Thick green is a track that is old enough to be counted and has a point. Thick red is also old enough to be counted but does not have a point in the current frame.

There are multiple ways to count the number of groups and group members, which are often based on a cross-line approach. In the observed case, there is a main walking direction along the *x*-axis of the image. However, it is possible to enter and leave the monitored area from all directions. Therefore, we count the members of the group when the group track is deleted, if it has been tracked for a minimum number of frames. This ensures that noise observed only in few frames will not be counted. Furthermore, the number of group members is filtered throughout the track, which ensures that the count is based on as many estimates as possible. The filtered estimate of group members is a floating point number; thus, the number is rounded off to the nearest integer when it is added to the count. Figure 5 shows an example of the running system and an explanation of the visual output.

## 5. Experimental Setup

The video material used is recorded by two thermal cameras of type AXIS Q1922-E [61], which supports a resolution of 640×480 pixels at 30 frames per second. A custom-built rig mounted on a light-post at a height of four meters is used to keep the cameras approximately parallel. The region observed has an area of approximately seven by six meters. The baseline should be as high as possible, keeping the dead distance (the distance to the closest point that both cameras are able to see) in mind, in order to achieve the best depth resolution. However, due to both the practical issues of mounting of the cameras as well as challenges with matching point correspondences with a large baseline, the cameras are mounted with a baseline of 0.7 m. The weather conditions are described as windy and sunny and 20 degrees Celsius, which causes the asphalt to be warmer than most of the pedestrians. The video materiel was recorded in August 2015 at the Tall Ships Races event in Aalborg, Denmark, an event that drew thousands of spectators to the harbour front. Thus, the video coverage is of a real-life scenario with pedestrians of varying ages and appearances in moderate densities with a high degree of occlusions. Three video samples of 5 min each were extracted. One of them was used for training, while the other two were used for testing. These are publicly available for download (http://www.vap.aau.dk/stereo-thermal-dataset/).

Ground truth is achieved by manual annotation according to the following rules: Individuals are counted if they are observed in at least 50 consecutive frames, even if they are heavily occluded. In general, if a human is able to recognize a pedestrian, so should the system. This rule also applies to children in prams and people in wheelchairs or on electric scooters. Since the top region of the street is not fully covered in the image, pedestrians are only counted if at least both shoulders are visible. For the bottom of the image, the head must be visible to be counted. Following these rules, ground truth is found for both test videos to be 174 and 215 pedestrians, respectively. With use of the training sequence the parameters of the system are tuned to: Learningrateofbackgroundmodel=0.998, $Eps_{\mathrm{group}}=0.7$ m, $MinPts_{\mathrm{group}}=10$, $Eps_{\mathrm{pedestrian}}=0.1$ m, $MinPts_{\mathrm{pedestrian}}=5$, Maximumdistancefrompredictedposition=0.8 m.

## 6. Results and Discussion

The performance of the system is tested on two thermal video sequences of 5 min each. The main result is the counting capability of the system, which compares the system count with the ground truth. In sequence 1, the system counts 166 pedestrians, while the ground truth states 174 pedestrians, resulting in a success rate of 95.4%. In sequence 2, the system counts 213 compared to the ground truth of 215, a success rate of 99.1%. These results show the counting system to be very robust.

To better understand the system’s performance, both video sequences are also evaluated using the number of true/false positive/negatives. A true positive corresponds to a correctly counted pedestrian. Likewise, a false negative corresponds to a missed pedestrian. False positives can be caused by three things: either a pedestrian is counted as two; pedestrian loses his/her tracker midway and is counted twice when the tracker is picked up again; or, if the system counts a pedestrian at the edge of the image, which should not happen according to the ground truth rules stated in Figure 5.

The results are summarized in Table 2. The sensitivity reveals the percentage of pedestrians that were counted, leaving out pedestrians counted twice. Precision refers to how certain the system is that a counted pedestrian is an actual pedestrian. A sensitivity percentage that is lower than a precision percentage indicates a tendency to count too low. The two effects, however, almost cancel each other out, which results in a high success rate despite the challenging real scenario. As one can see, the first video sequence contains more children, who are at risk of not being counted since fewer of their features are identified due to their height. This is the main reason for the lower percentages and higher number of false negatives in sequence 1 than in sequence 2.

**Table 2:** Performance results for the two 5 min test videos.

	Sequence 1	Sequence 2	Formula
**Ground truth**	174	215	
**True Positives**	147	194	
**False Positives**	19	19	
**False Negatives**	27	21	
**Sensitivity**	84.5%	90.2%	TP/(TP + FN)
**Precision**	88.6%	91.1%	TP/(TP + FP)
**Accuracy**	76.2%	82.9%	TP/(TP + FP + FN)
**Success rate**	95.4%	99.1%	(TP + FP)/(TP + FN)

**Figure 6:**
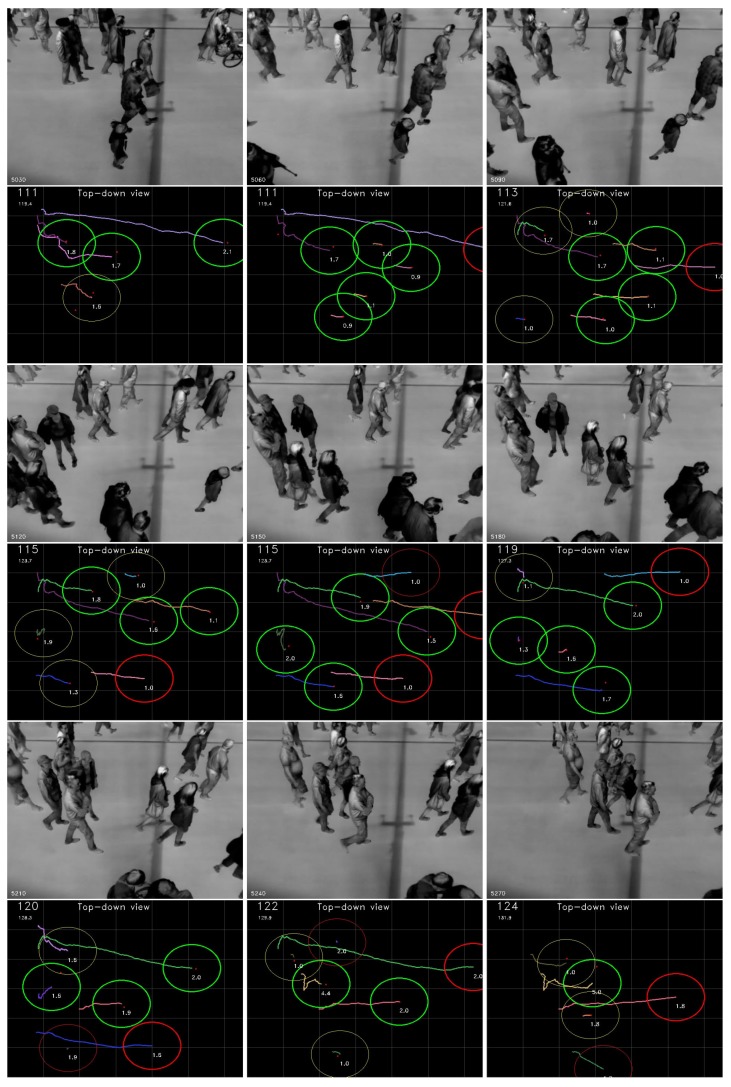
Each image has 30 frames of one second duration. The systems prediction of pedestrians is generally accurate despite the moderate density and high degree of occlusions of real data.

Frames from an eight second sequence are included in Figure 6 to demonstrate the system in a typically occluded scenario. The occlusion is especially evident in the first 4–5 images where the person starting in the top left corner almost fully covers the person behind. Due to the clustering approach the system predicts that the cluster will contain 1.5 pedestrians, which is rounded to two. The predicted number of pedestrians are generally close to the actual number throughout the scene.

No direct comparison of results can be made since no public available stereo thermal database was found. Furthermore, studies that uses popular datasets, such as PETS 2009 [62], evaluates crowd estimation on a frame-by-frame basis using the two measures Mean Absolute Error (MAE) and Mean Relative Error (MRE). As a result, they do not count the number of pedestrians passing by; rather, they count the number of pedestrians present in the scene. Because the measure is not directly transferable to the number of pedestrians that have walked through the scene, we do not compare our method to any crowd estimation method.

A system with a comparable viewing angle, but using stereo RGB cameras, achieved 89.29% accuracy in a scene with pedestrians moving in multiple directions [41]. A few comparable systems used a stereo camera or Kinect sensor with a zenithal view to avoid difficult occlusions. One of these systems was tested on a similarly challenging scenario, counting pedestrians attending a carnival [63]. Here, an error mean of 5.8% was achieved, which is slightly higher than ours. Tests on more simple scenarios, also captured from a zenithal view, are presented in [64,65], and both report accuracies around 99%.

The method described in this work is robust to changes in the setup. This is mainly due to the use of the segmentation using an adaptive background model, the translation, scale, and rotation invariant SIFT descriptors, and the hierarchical 3D clustering based on proxemics theory. However, the movement patterns of pedestrians might change between different scenes. This means that the parameters of the Kalman filters have to be fine-tuned for different scenarios. Also, the distances between pedestrians will vary from the guidelines defined by Hall [55] in very crowded scenarios. Thus, this method is not directly applicable to dense scenes without changing the rules of group definition. Furthermore, the transformation from camera space to street space is setup specific and has to be defined for different setups.

In practice, the requirements for a stereo setup are more demanding than for a monocular setup. 3D reconstruction is only achievable in the field of view shared by the two cameras. For which reason the cameras have to be carefully placed in relation to each other and the observed area. Currently, there are no factory calibrated stereo thermal cameras on the market. Therefore, two individual thermal cameras have to be used, which requires stereo calibration to relate the two camera views. A process that can be challenging in the LWIR spectrum compared to the visible spectrum.

## 7. Conclusions

This paper demonstrates the use of thermal cameras in the application of pedestrian counting. A study of features in thermal images shows the best results when using a Shi–Tomasi detector with SIFT descriptors. A stereo setup is utilized to handle occlusions by obtaining sparse 3D points and grouping these by the DBSCAN clustering algorithm. Each cluster is tracked by a Kalman filter for robust counting. This approach is tested in a real-case scenario with moderate densities and heavy occlusions, showing that thermal cameras are able to compete with RGB-camera accuracies, making it lucrative to use them in public places both night and day.

Future work should include a comprehensive study of how the stereo thermal modality performs in a variety of scenarios caused by weather and densities of pedestrians. Moreover, the stereo thermal camera method should be analyzed in-depth in terms of other common modalities of pedestrian counting, which would allow the authors to qualitatively as well as quantitatively compare the method to current state-of-the-art methods. Finally, future studies could include more complex scenes in which multiple setups are used to monitor several areas or even applications with other objects of interest, such as animals.
